# A step towards valid detection and quantification of lung cancer volume in experimental mice with contrast agent-based X-ray microtomography

**DOI:** 10.1038/s41598-018-37394-w

**Published:** 2019-02-04

**Authors:** Pidassa Bidola, Juliana Martins de Souza e Silva, Klaus Achterhold, Enkhtsetseg Munkhbaatar, Philipp J. Jost, Anna-Lena Meinhardt, Kirsten Taphorn, Marie-Christine Zdora, Franz Pfeiffer, Julia Herzen

**Affiliations:** 1 Department of Physics & Munich School of Bioengineering, Technical University of Munich, 85748 Garching, Germany; 2 III. Medizinische Klinik, Klinikum rechts der Isar, Technical University of Munich, 81675 Munich, Germany; 3 Diamond Light Source, Harwell Science and Innovation Campus, Didcot, Oxfordshire OX11 0DE United Kingdom; 4 Department of Physics & Astronomy, University College London, London, WC1E 6BT United Kingdom; 5 Department of Diagnostic and Interventional Radiology, Klinikum rechts der Isar, Technical University of Munich, 81675 Munich, Germany; 6 Present Address: Institute of Physics, Martin-Luther-University Halle-Wittenberg, 06120 Halle (Saale), Germany

## Abstract

Tumor volume is a parameter used to evaluate the performance of new therapies in lung cancer research. Conventional methods that are used to estimate tumor size in mouse models fail to provide fast and reliable volumetric data for tumors grown non-subcutaneously. Here, we evaluated the use of iodine-staining combined with micro-computed tomography (micro-CT) to estimate the tumor volume of *ex vivo* tumor-burdened lungs. We obtained fast high spatial resolution three-dimensional information of the lungs, and we demonstrated that iodine-staining highlights tumors and unhealthy tissue. We processed iodine-stained lungs for histopathological analysis with routine hematoxylin and eosin (H&E) staining. We compared the traditional tumor burden estimation performed manually with H&E histological slices with a semi-automated method using micro-CT datasets. In mouse models that develop lung tumors with well precise boundaries, the method that we describe here enables to perform a quick estimation of tumorous tissue volume in micro-CT images. Our method overestimates the tumor burden in tumors surrounded by abnormal tissue, while traditional histopathological analysis underestimates tumor volume. We propose to embed micro-CT imaging to the traditional workflow of tumorous lung analyses in preclinical cancer research as a strategy to obtain a more accurate estimation of the total lung tumor burden.

## Introduction

Over the years, researchers have benefited enormously from genetically engineered mouse models for lung cancer^1–4^. These models closely resemble the human pathology^5,6^, providing a deep understanding of the steps involved in the progression of the disease. Additionally, these mouse models are used to study the response and resistance to innovative therapies to treat cancer in studies of lung carcinogenesis with experimental mice^1,5–10^.

As the tumor volume reflects the progression or regression of cancer^11^, tumor size is the main parameter used in cancer model studies to evaluate the success of a therapy^12,13^. In the case of subcutaneous xenograft tumors, the volumetric estimation is traditionally done by standard external caliper measurements of the tumor dimensions^14,15^, which is a simple, fast and well-established approach. However, unlike the subcutaneous xenografts, the lung tumors grow non-subcutaneously in genetically engineered mouse models^13^, and are clearly not compatible with caliper measurements.

Histologic analysis is a well-established approach used in the clinics for the examination and diagnosis of cancer types in diseased lung tissues, and it is the standard tumor size measuring method when caliper measurements fail. Histopathological investigation of the tumorous lungs is done *ex vivo*^5,13^ and tumor burden is determined by the percentage of total lung area occupied by the tumorous tissue in a histologic slice^2^. Histopathology involves tedious time-consuming manipulation of the specimen, which is cut into thin slices, stained and inspected in an optical microscope. Optical microscopes display high-contrast and high-resolution images in two-dimensions (2D) and, ideally, the digitization of several 2D histological sections would provide a three-dimensional (3D) histopathological image of the entire specimen, that would allow the quantification of tumor volume. However, 3D reconstruction of an entire volume from histological data presents drawbacks, mostly related to the tissue deformation inherently induced by the manipulation of the sample during the preparation process^16–19^. Therefore, until now, histopathology still cannot produce knowledge of the total tumor volume and it is only able to provide a few spatially limited, two-dimensional images of a three-dimensional structure. Traditionally, tumor burden is calculated from a few histological cuts of the pathologic specimen and, for that reason, tumor studies would benefit from a more precise quantification of tumors’ volume. It requires an imaging modality capable of representing the pulmonary architecture in 3D, as well as detecting the tumorous lesions. Determining the tumor volume of internal cancers is challenging and only few researchers have been working on establishing reliable methods to perform these measurements *in vivo*^2,5,12,15,20–26^. However, no definitive method for tumor burden evaluation has been described yet.

Very recently, staining of soft-tissue samples combined with X-ray absorption micro-computed tomography (micro-CT) has been demonstrated to be a beneficial tool for imaging soft-tissue biological specimens^27–31^. This method has been mostly applied to anatomical studies^30–33^ and provides non-distorted 3D highly-resolved micro-CT images of organs or parts of mammals^30,34^. In this work, we explored staining and micro-CT to visualize *ex vivo* lungs and to quantify the volume of tumors in genetically engineered mouse models of pulmonary cancer. After performing tests on healthy lungs of wild-type mice using some common staining agents already explored in micro-CT, we selected an appropriate staining protocol for this organ and proceeded with further investigations of the tumor-burdened counterparts. We used a semi-automated method to perform 3D segmentation of the abnormal areas in the reconstructed micro-CT data of the scanned tumor-burdened lungs. From this analysis, we could perform real volumetric calculation of the ratio of abnormal tissue in the total lung tissue volume. We applied our method to a total of four pathogenic specimens and we discuss its advantages and limitations for tumor bulk evaluation comparing it to the histopathological tumor burden analysis performed with hematoxylin and eosin-stained (H&E) slices.

## Methods

### Contrast agents

For our experiments, we adopted the staining protocols presented in Metscher *et al*.^29^. Three solutions were used: (1) phosphotungstic acid in water (PTA); (2) a mixture of iodine (I_2_) and potassium iodide (KI) in water (named as IKI); and (3) iodine dissolved in absolute ethanol (I2E).

### Sample preparation

Prior to the main experiment, four non-tumorous lungs stained with different contrast agents were analyzed to determine the best contrast medium for this study. The size of the lungs ranged between 1.2 and 1.3 cm in diameter (Supplementary Fig. 1). In a preliminary preparation, the lungs were inflated preceding the closure of the trachea by a surgical suture. They were fixed in 4 % paraformaldehyde and subsequently placed in 70 % ethanol solution in order to shrink the lungs to a stable size^35^. The staining was executed by placing each lung in one of the following contrast agent solutions for 14 h: PTA, IKI or I2E. One lung used as control was not stained and was solely set in 70 % ethanol aqueous solution. Afterwards, the lungs were washed and placed in a tube filled with 70 % ethanol solution for the imaging experiment.

The investigation of tumorous lungs was performed *ex vivo* on specimens taken from mice after 19 weeks of tumor induction. At an age of six to eight weeks, the mice were infected intranasally with a Cre-expressing adenovirus, which results in the Cre-mediated excision of a gene from its genetic recombination site called “STOP cassette”. This allows the tissue-specific genetic expression of the formerly silent K-Ras^G12D^ gene, a pro-proliferative gene carrying the G12D-mutation to turn into a tumor-driver^36^. As this gene is often mutated in humans, this is not only a well-established mouse model, but also a good representation of the disease in humans. In addition to possessing the driving mutation, the mice used in our imaging experiments could further be divided into Mcl-1^+/+^, Mcl-1^fl/+^ and Mcl-1^fl/fl^ genotypes^37^. By the same mechanism, the Cre-enzyme leads to the removal, and thus, deficiency, of one or both alleles of this gene.

### Micro-computed tomography protocol

The commercial laboratory micro-CT system ZEISS Xradia 500 Versa was used for the measurements presented here. It is equipped with a tungsten X-ray micro-focus source with a spot size of up to 3 μm (at 90 kV, 80 μA), that allows the minimization of focal spot blur and improves the spatial resolution. The detector is a cooled CCD camera of 2048 × 2048 pixels, coupled to various switchable-objective lens units. The size of each pixel is 13.5 μm. All measurements were performed at a tube voltage of 40 kV and a current of 63 μA using the 0.39x objective lens. The source-to-sample and sample-to-detector distances were chosen individually for each specimen in order to position the entire sample in the optimal field of view. Consequently, the effective pixel size differed slightly among scans, but was always in the range of 12 to 13 μm. A total of 1201 projections were acquired over 360 degrees in each scan. A binning of 2 × 2 pixels was used to enable an image acquisition with a reasonably short exposure time of 12 s per projection, whereas the image reconstruction was performed without binning. Voxel size in the final micro-CT datasets is in the range of 12 to 13 μm.

### Visualization

The 3D rendered images shown here were obtained from data reconstructed as 16-bit TIFF images and processed with the software VGStudio Max (Volume Graphics GmbH, Heidelberg, Germany). Thereafter, segmentation was performed to differentiate the tumors from the normal lung tissues. For an accurate volume extraction of the tumorous areas, the semi-automated tool “region grower” available in VGStudio Max was used. This tool selects a region with a flooding algorithm. The “static mode” option adds a voxel if it has a gray value that varies no more than half of the adjusted tolerance of gray value range. For more accuracy, the “region grower” tool provides the adjustment “radius”, that restricts the area of the flooding algorithm to a sphere with a certain radius. Therefore, the selection of the trachea can be prevented.

### Ethics statement

Animals were maintained under pathogen-free conditions, and all animal experiments were conducted in compliance with protocols approved by the animal ethics committee guidelines of Regierung von Oberbayern (Ethics number AZ: 55.2-1-54-2532-55-12).

## Results

### Staining agent selection

**Figure 1:**
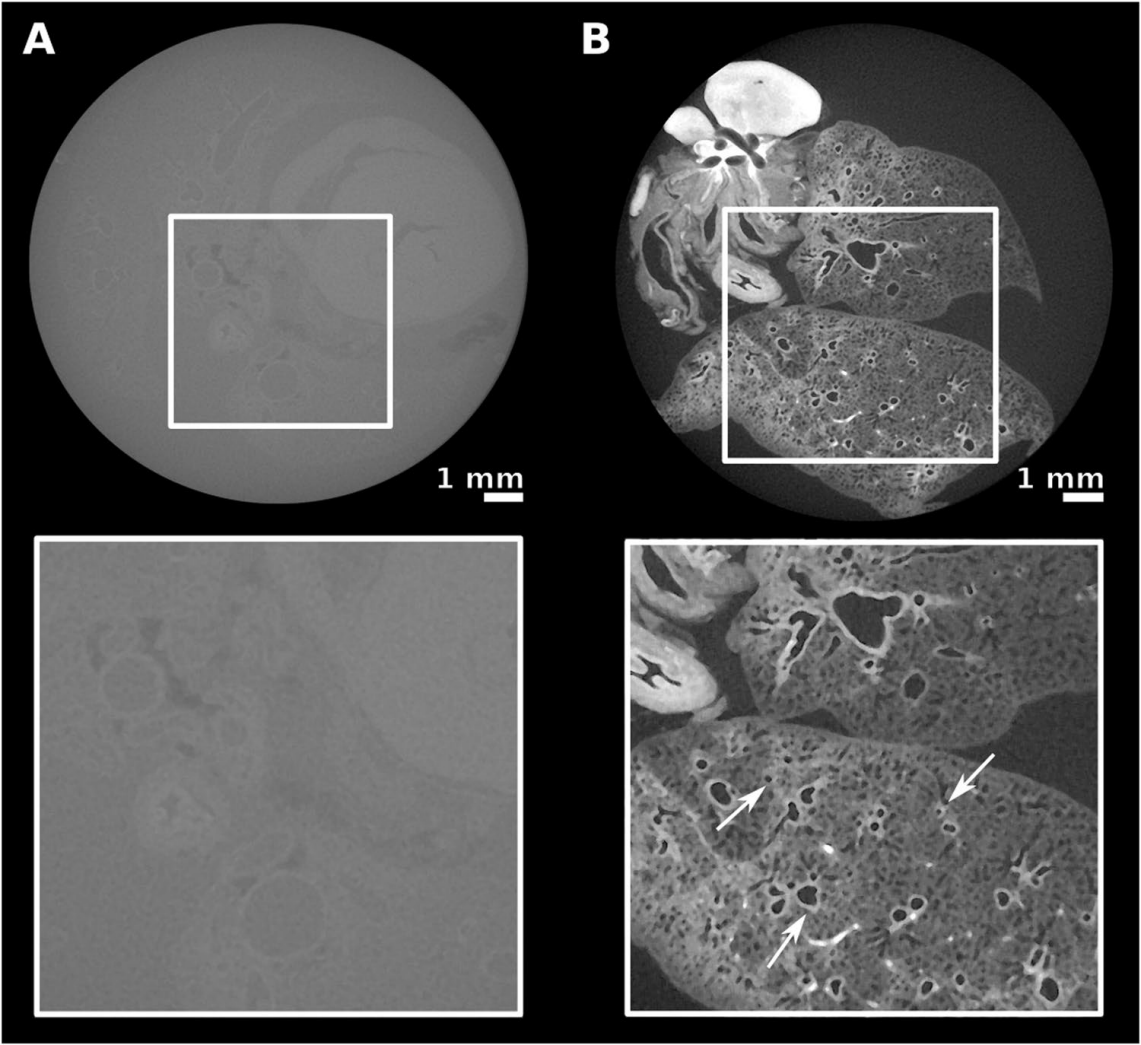
Comparison between tomograms of unstained and stained lungs. The staining procedure with I2E increased the image contrast, as is seen in the comparison of the tomograms of (**A**) an unstained sample and of (**B**) a lung stained with I2E. Details are better seen in the enlarged images of the areas selected with a square, shown below the corresponding sample. Arrows indicate lobe bronchi and small airways.

A comparison of the images of an unstained lung with a stained one, both imaged by micro-CT under the same experimental conditions, highlights the need of using a contrast agent to enable the visualization of well-resolved structures in the sample (Fig. 1). In the unstained lung tomogram (Fig. 1A), the gray values of the lung tissue and ethanol do not differ considerably, making any precise description of the pulmonary structures extremely difficult. A comparison of the image contrast obtained after applying the staining agents tested here (Fig. 1B and Supplementary Fig. 2) shows that the best results were obtained after using a solution of iodine in ethanol (I2E). With this staining agent, images with high-contrast were obtained. When PTA was used as staining agent for the lungs (Supplementary Fig. 2A), a good image contrast was obtained; however, PTA seems not to diffuse homogeneously into the sample, accumulating on the surface of the organ. When IKI was used as staining agent for the lung (Supplementary Fig. 2B), the differences in absorbance levels between different parts of the organ were not quite as high as those observed with I2E. I2E diffused homogeneously through the connective tissue of the lung and bound to its vascular walls and, therefore, the finest features of the lung were clearly highlighted. Indeed, lobe bronchi as well as the terminal bronchioles, were well resolved (Fig. 1B). Compared to the other staining agents tested here, I2E significantly improved the image contrast within the lung structures and was then used in the following investigations of the tumorous lungs.

### Tumorous area segmentation and volume estimation

**Figure 2:**
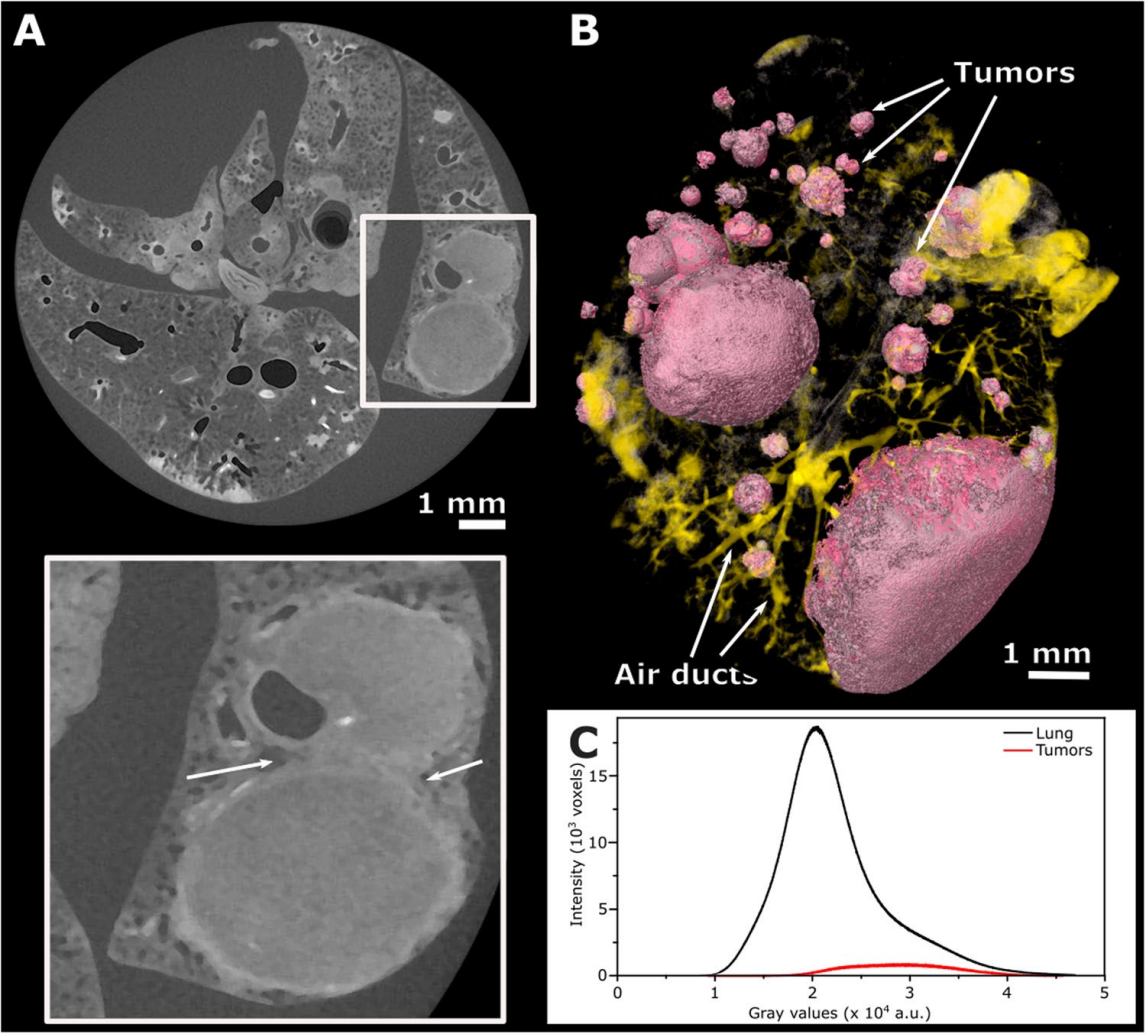
Images of a tumorous lung. (**A**) Tomogram of a tumorous lung. The area selected by the square was enlarged to show a tumorous area and the undefined boundaries between two tumors. The excellent contrast obtained for lesions (due to I2E-staining) and airways allowed their segmentation and reconstruction shown in (**B**), where tumorous areas (light pink) and airways (yellow) are well discriminated from the healthy soft-tissue, rendered colorless, and therefore not visible. Distribution of voxel intensity values for the whole organ and for the total volume of tumors in this sample (**C**) shows that tumorous lesions correspond to 15.4 % of the entire lung’s volume.

**Figure 3:**
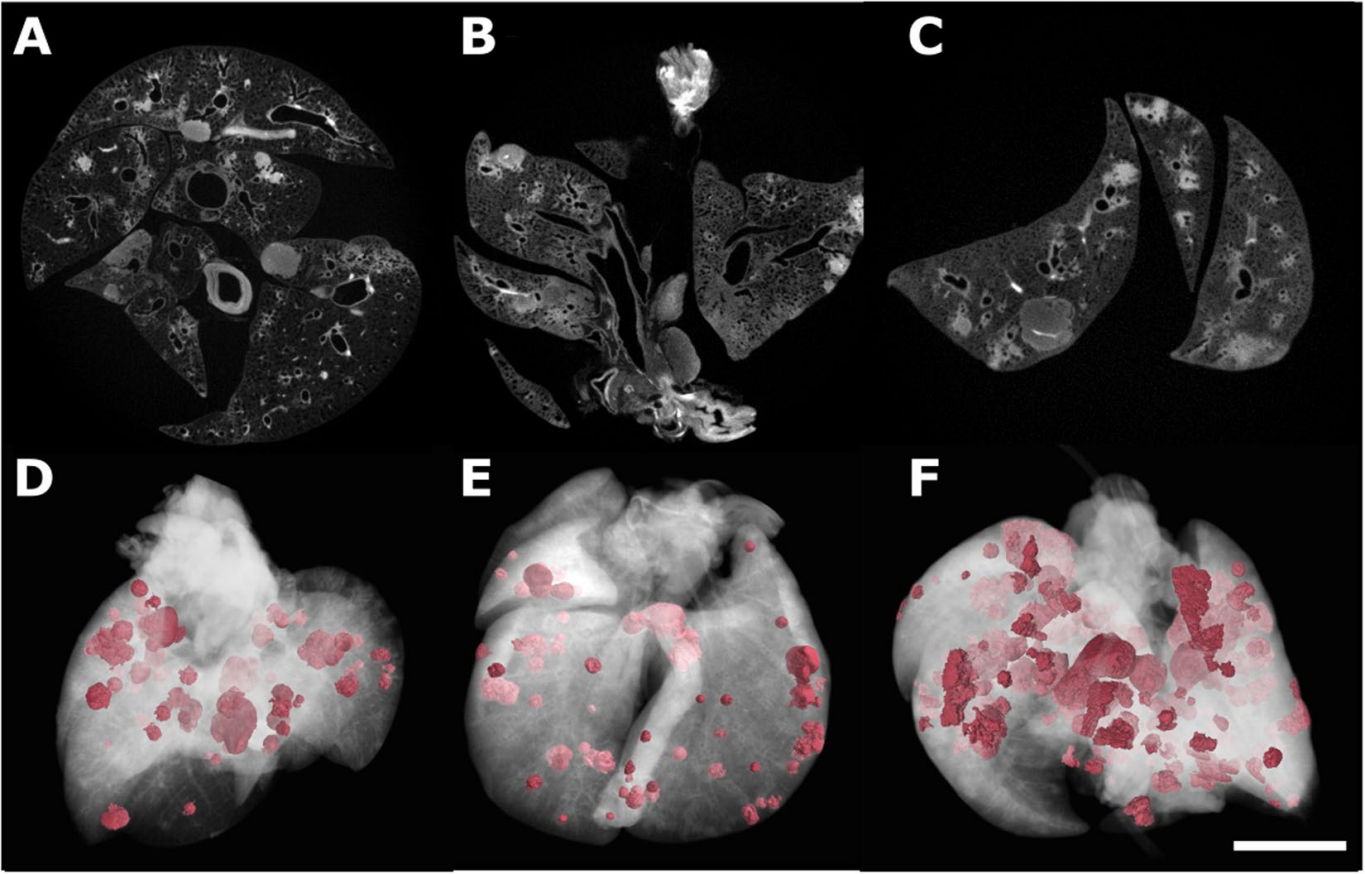
2D and 3D images of three different I2E-stained tumorous lungs. Tomograms of three tumorous lung samples (**A**–**C**) with their corresponding three-dimensional renderings (**D**–**F**) shown below, with normal lung tissue represented in transparent white to allow the visualization of the tumorous lesions pseudo-colored in pink. Pink regions in (**D**) correspond to 16.9 % of the total volume of this specimens, while this number is equal to 20.7 % in (**E**), and 65.8 % in (**F**). Scale bar: 1 mm.

**Figure 4:**
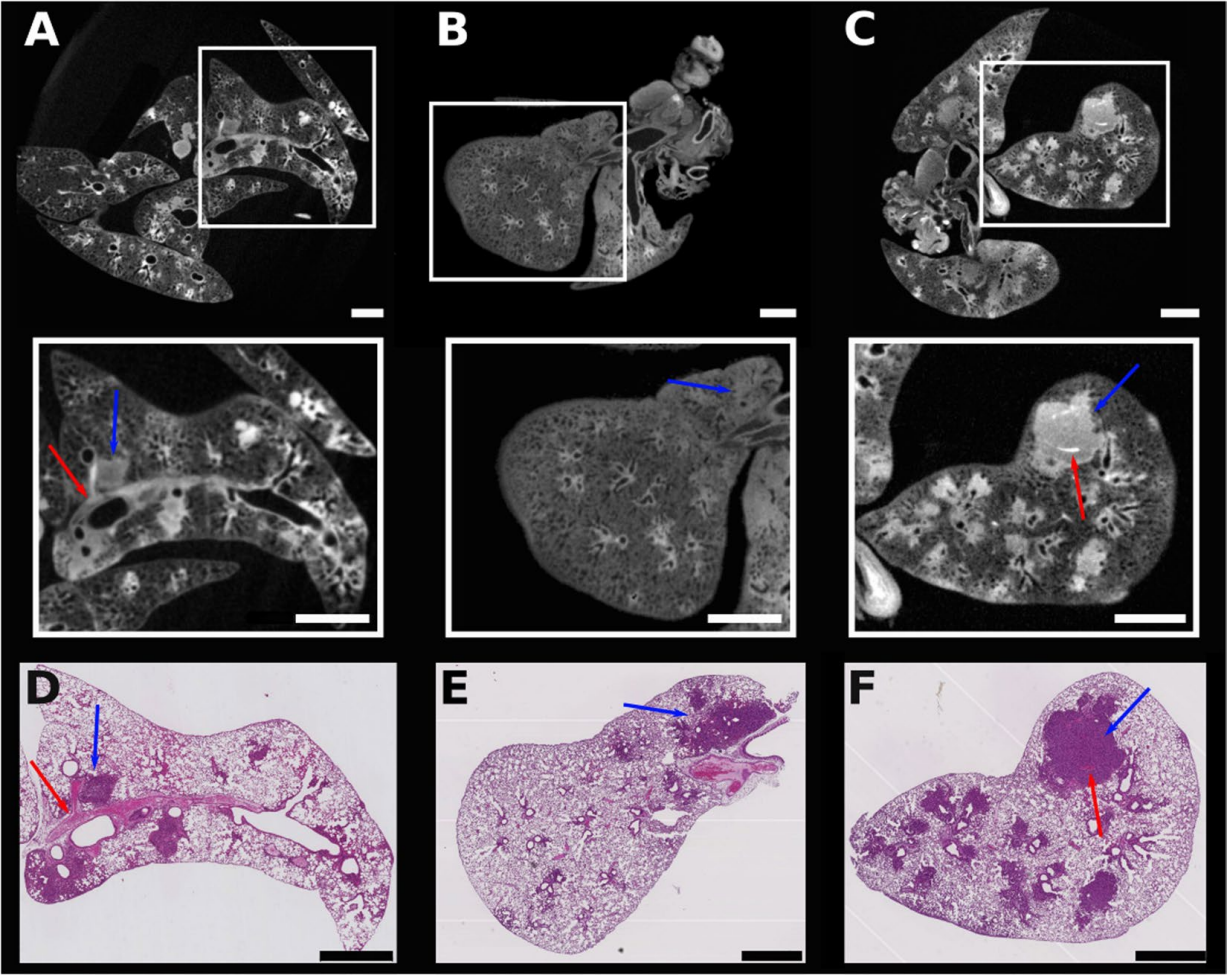
Tomograms and comparable H&E-stained histological sections of three tumorous lungs. Tomograms of three different tumorous lungs (**A** to **C**), with the enlarged areas equivalent to the histological slices (**D**–**F**). Blue arrows point to tumor, while red solid arrows indicate blood vessels. Tumor burden calculated from the images are shown in Table 1. Scale bar: 1 mm.

When applied to a diseased lung, we confirmed that I2E-staining not only spots the main structures of the organ, but also highlights the tumorous areas (Fig. 2A). The image’s contrast obtained when this staining agent is used, as well as the air present in the lung airways, allows the segmentation of both tumors and air ducts from the surrounding tissue (Fig. 2B). The segmentation procedure consists of selecting only the range of gray values that corresponds to that specific tissue. Therefore, it is possible to visualize the tumor and also to estimate its size by calculating the ratio of voxels related to the tumorous lesions to the overall number of voxels of the entire specimen imaged. We used a semi-automated tool of the software VGStudio MAX known as “region grower” to evaluate the percentage of tumors that have grown within defined boundaries in the organ. Using this tool, tumors of different shapes and sizes were segmented from the specimen (Figs 2B and 3D–F). For the quantification of the tumor burden, a histogram containing the distribution of grey values of the micro-CT volumetric image was generated and the segmented part was also expressed in terms of gray value intensities. This method of estimating the volume of the tumorous lesions is illustrated here for one specific lung (Fig. 2C). The tumorous regions were segmented in the micro-CT image and pseudo-colored in pink (Fig. 2B), while the entire volume of the lung was rendered transparent to enable proper visualization of the segmented parts. In the histogram (Fig. 2C), the total gray values and the gray values corresponding to the pink areas are displayed separately, and the pink areas correspond to 15.4 % of the volume of this lung specimen. This same analysis was performed on three additional tumorous samples stained with I2E (Fig. 3, Table 1). The total volume of tumorous tissue calculated is equal to 16.9, 20.7 and 65.8 % of the overall volume of the lung specimens illustrated in Fig. 3D,E and F, respectively. In Figs 3 and 4, lungs from mouse of the same genotype Mcl-1 are shown (Fig. 3A and Fig. 4A: Mcl-1+/+, Figs 3B and 4B: Mcl-1 fl/+ Figs 3C and 4C: Mcl-1 fl/fl).

**Table 1:** Tumor burden calculation performed for different samples using three different methods: (1) from the tumor area segmented manually in 2D images of H&E-stained specimens; (2) from the tumor area segmented using a semi-automated method and micro-CT slices of I2E-stained specimen; and (3) and tumor volume reported as the sum of measurements of all the tumorous areas of all virtual micro-CT slices.

Sample	Tumor burden (%)		
	H&E histological slice	micro-CT virtual slice	micro-CT volumetric image
1^a^	6.9	7.6	16.9
2^b^	5.9	—	20.7
3^c^	20.5	22.8	65.8
4^d^	3.3	10.5	8.8

**Figure 5:**
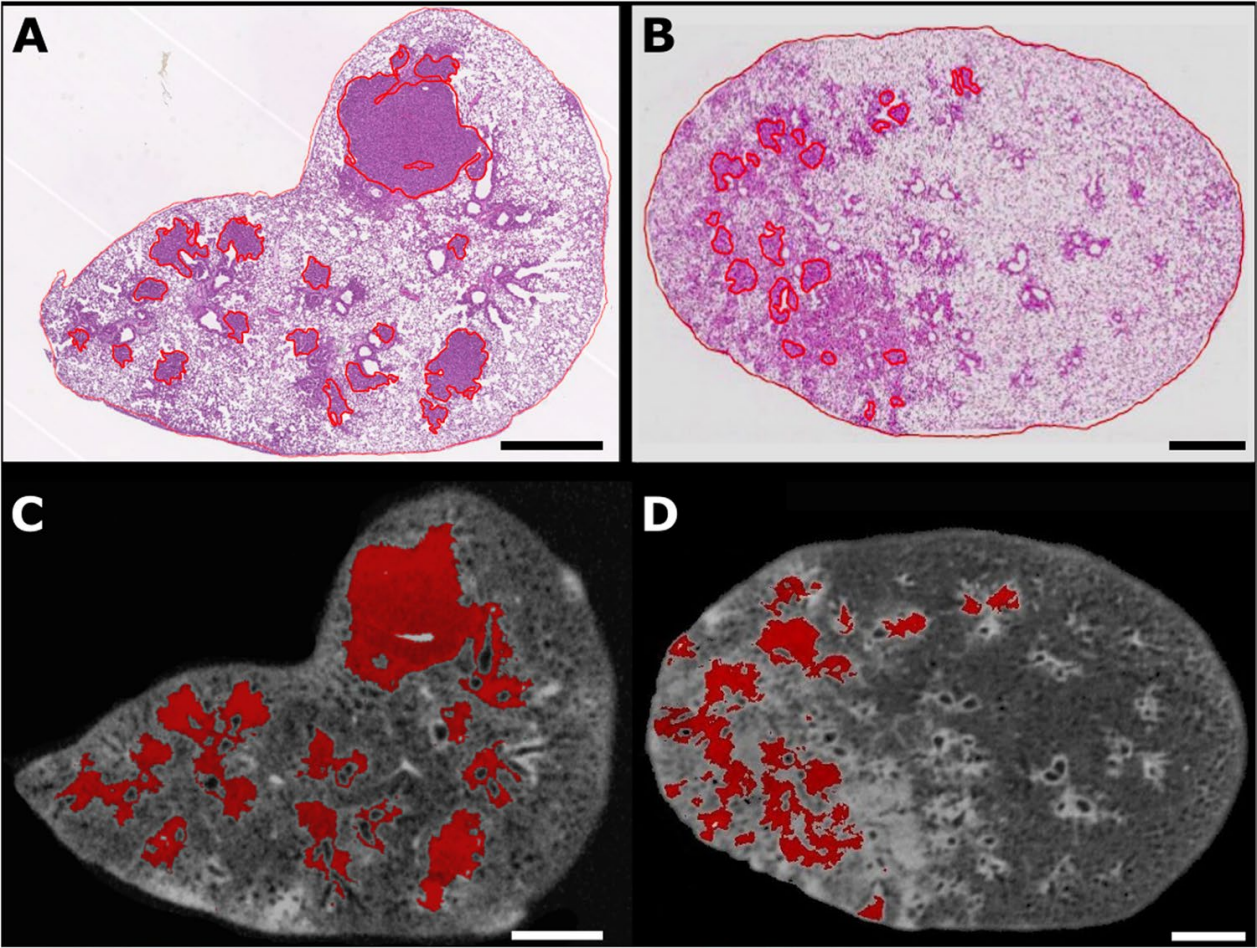
Segmentation of tumorous areas in histological sections and their more resembling virtual micro-CT slices. Histological sections of diseased lungs with the tumorous areas delimited by red lines (**A**,**B**) and the corresponding virtual micro-CT tomograms shown below (**C**,**D**). Red marked areas in the virtual slices correspond to tumors identified using the semi-automated “region grower” tool of VGStudio Max. Tumor burden calculated from the images are shown in Table 1. Scale bar: 1 mm.

Columns from left to right: ^a^Figs 3D and 4A,D and ; ^b^Figs 3E and 4B,E; ^c^Figs 3F and 4C,F (respectively equal to Fig. 5A,C); ^d^Fig. 5B,D, and micro-CT volumetric image not shown.

### Validation of the method for tumorous lesions volume estimation

The tumorous lungs were first inspected by micro-CT, then later processed for histopathological analysis. They were embedded in paraffin, sliced, stained with hematoxylin and eosin (H&E) and then imaged with an optical microscope. Virtual slices obtained from micro-CT data and similar H&E-stained slices of the same sample were compared (Fig. 4). The contrast of the images at high-spatial resolution allows differentiating the lesions from the healthy tissue areas in both lung’s virtual and histological slices (Fig. 4A–F, respectively). Almost identical structures are observed in the slices stained with H&E (Fig. 4D–F) and the analogous virtual micro-CT slices (Fig. 4A–C, enlarged areas), as indicated by the arrows that identify tumor (blue) and blood vessels (red). We evaluated the tumors from the micro-CT virtual slices using the semi-automated method described above and we compared these results with a similar estimation performed by an experienced pathologist on the histological slices. For one of the samples, the tumor cross-sectional area is equal to 7.6 % when this value was calculated from the micro-CT tomogram (Fig. 4A, zoomed area), and 6.9 % when it was estimated from the histopathological slice (Fig. 4D). Similarly, 22.8 % versus 20.5 % were the numbers obtained from the calculations performed in Fig. 4C,F, respectively. The low quality of the image contrast in Fig. 4B limited the accuracy of the calculation and it is not shown here.

To understand the differences obtained in the tumor burden calculated using the histological and virtual slices presented here, a comparison between the tumorous areas marked in histological slices and the nearly equivalent micro-CT virtual slices is necessary. The tumorous regions marked with red lines in the histological slices in Fig. 5A,B were delineated after visual analysis performed by an experienced pathologist. They correspond to 20.5% (Fig. 5A) and 3.3% (Fig. 5B) of the total surface of the lung in these specific slices. Other areas located in the outer limit of the red contours, although stained in a darker pink tone, do not correspond to tumors, but to abnormal tissue, such as dead and/or stressed tissue due to inflammation or pre-stages of tumor development. The semi-automated segmentation tool used to define the tumorous areas in the virtual micro-CT slices gives slightly higher numbers and the areas marked red in Fig. 5C,D are equal to 22.8 % and 10.5 %, respectively.

## Discussion

In this study, we evaluated the use of contrast agent-based X-ray microtomography for the quantification of lung cancer volume in experimental mice. For that, we chose a well-established mouse model of pulmonary cancer, which is also a good representation of the disease in humans^5,6^. We then tested some staining agents among a few well known in the literature, that are used to increase the contrast of soft-tissue in X-ray absorption-based micro-CT^28,29^. The need for using a contrast agent in soft-tissue micro-CT imaging is exemplified in the comparison of a non-stained lung (Fig. 1A) to a stained one (Fig. 1B). We tested staining solutions containing either tungsten or iodine as X-ray absorbing elements (Fig. 1B and Supplementary Fig. 2), and we finally selected iodine-in-ethanol (I2E) solution as the best staining agent for our purposes. The I2E solution is known as a good contrast agent to highlight the morphology of mammals’ organs^28–30,34,38^, including the lungs^34^. As we show here, I2E staining is an adequate method to distinguish unhealthy from healthy tissue in the diseased lungs imaged with micro-CT (Fig. 2). Indeed, micro-CT imaging of I2E-stained specimens reveals large and small tumors, and tumors with different morphologies (Fig. 2B). Due to the small voxel size obtained in the 3D reconstructed I2E-stained lungs imaged with micro-CT (ca. 13 μm), we were able to finely segment the tumors that grow with defined boundaries. We evaluated the tumor burden using a semi-automated method, that uses grey-level threshold and region-grow algorithms to segment out the anatomic structures of interest (the tumorous regions) in the 3D micro-CT images. To validate the method of tumor burden estimation that we are proposing here, we compared the results of tumor burden estimated by the micro-CT images using this semi-automated method to segment the tumorous tissue, and the histological assessment of tumor burden using H&E-stained slices performed by an experienced pathologist. Therefore, the tumorous lungs that were previously scanned with micro-CT were later submitted to histopathological analysis with routine H&E-staining. Here we show that the I2E stain used to generate contrast in the micro-CT images is compatible with further routine H&E-staining, and the histopathological analysis of the tumorous lungs stained twice, first with I2E, then with H&E, was performed without any significant issue.

When comparing the size of the total tumorous regions estimated using micro-CT virtual slices and H&E-stained slices, there is a divergence in the percentage of tumorous areas calculated (Fig. 5A,B *vs*. Fig. 5C,D, and Table 1). Even considering small deviations conditioned by the individual interpretation of suspicious structures in the histological slice, the numbers obtained in the micro-CT image-based calculations are consistently slightly higher than those obtained in the histopathological studies (Table 1). These results might be due to the fact that the stain used here to improve the contrast in micro-CT (I2E) is non-specific to tumorous tissue^39^. As a result, similar gray-scale values are obtained for tumor, dead tissue, and tissue with inflammation. Thus, due to the limited contrast resolution, they cannot be distinguished by our proposed semi-automated method, which then also takes into consideration in the tumor size estimation the dead tissue and the tissue with inflammation. It does not invalidate our proposed method because the finding of interest in cancer research is often the sum differences between two groups of mouse models: the wild-type and the mutated-type^23^. However, this effect must be taken into consideration mainly when studying mouse models that use the intranasal adenoviral infection to induce tumor development. The infection leads not only to the formation of multifocal small tumors with irregular shapes^2^, but also to inflammation of the lung tissue^40,41^. On one hand, our method is adequate to detect these multifocal small tumors, which might not be easily visualized in histological analysis, but on the other hand, it also detects other unhealthy tissues, and all are considered in the final tumor burden evaluation, resulting in larger tumor burden estimated by the micro-CT data. Another source of discrepancy in the numbers calculated using histology and micro-CT data is the modification of specimen size during the sample processing for histology. It has been shown that shrinkage and deformation occur during histological processing of the specimen, leading to a significant volume reduction^23,42^. The I2E-staining procedure used for micro-CT is known to cause shrinkage of the specimen due to its dehydration^30^; however additional reduction of the specimen size cannot be excluded and might occur due to the further H&E-staining performed in the specimens analyzed here (Table 1).

A limitation of our method related to the detection of tumors with irregular shapes is noticeable when analyzing the micro-CT data. The growth of tumors not always occurs with well-defined boundaries and burdens may not have very precise edges. As exemplified in the region indicated by the arrows in the enlarged area in Fig. 2A, lesions may connect to each other and it is difficult to define the tumor boundaries. Indeed, during the evaluation of our proposed method, we observed that the regions covered by a tumor that has grown with undefined boundaries are a challenge for a precise semi-automated micro-CT image-based segmentation. This problem could, though, be corrected by visually inspecting the slices and manually adjusting the tumor contour before the final volumetric calculation.

Compared to *in vivo* micro-CT, that is being studied as an alternative to histology to evaluate tumor burden in lungs^2,23^, our *ex vivo* micro-CT method provides final images with higher spatial resolution, thus smaller tumors can be detected. Also, *ex vivo* micro-CT does not present the limitations related to image blurring due to respiratory and cardiac motion artifacts during image collection that are present in *in vivo* experiments^2,23,42^. Moreover, in *in vivo* micro-CT, images with inferior contrast are obtained since the use of contrast agents is limited to their short half-life in the mouse body^23,24^. Regarding the challenges to segment tumors with irregular shapes, both *in vivo* micro-CT and *ex vivo* of stained specimens share this same limitation^2^. In addition, though due to different reasons, tumor size evaluation done with *in vivo* micro-CT and our *ex vivo* micro-CT method consistently overestimates the tumor burden^42^.

H&E-staining is currently the gold standard in histopathological studies and plays a key role in studies of tissues for diagnosis and research purposes^43^. H&E-staining provides a detailed representation of tissues with high-resolution for histopathological studies, thus allowing the identification of tissue abnormalities. However, it is known that some differences in the evaluation of tumor extents are likely to occur even among experienced histopathologists. Moreover, histopathology does not allow the volumetric study of tumors, and evaluating the percentage of tumorous tissue based on a few histopathological slices presents a major drawback related to the missing information from the large number of excluded slices. On the other hand, we show here that, though the evaluation of the tumorous regions based on micro-CT data of I2E-stained lungs results in slightly overestimated tumor burden, it provides additional volumetric information that is not available when imaging histological slices using an optical microscope or any other 2D imaging technique. Furthermore, micro-CT not only allows the visualization of the sample through any virtual plane without the need of physically slicing the specimen, but it is also compatible with further H&E-staining and histopathological investigations. Also, here we do not restrict our analyses to one slice or to one specific lung lobe. For these reasons, performing micro-CT prior to H&E-staining would be advisable in a workflow, as the first does not prevent the later to be completed. Moreover, in the case of tumor volume studies, micro-CT would provide extra volumetric information that is important in the evaluation of potential differences in the growth patterns of different tumor types. Also, the semi-automated method proposed here is not limited to the VGStudio software. It is based on a grey-level threshold and is easily applied in the workflow of other 3D image processing software. It must be taken into account, though, that proper I2E staining and appropriate micro-CT imaging are necessary to obtain good-quality images for tumor burden quantification using the method that we are introducing here. We anticipate that the tumor burden evaluation method proposed by us, based on staining, micro-CT and semi-automated segmentation of tumors can easily be used in several research laboratories and would provide additional volumetric information at the final steps of longitudinal cancer studies.

In conclusion, here we show that the selection of an appropriate staining protocol combined with *ex vivo* micro-CT is an adequate method for studying tumorous lung tissue. Using I2E as staining agent, we obtained micro-CT images with high-contrast, leading to high-resolution 3D information, that enables the detection of tumors as small as a few mm^3^ and the inherent registration of the sample slices without the need of physically sectioning the sample. Especially in genetically engineered mouse models that produce lung tumors with well precise boundaries, the method that we present and describe here could be used to quickly evaluate the tumorous tissue volume. For tumors surrounded by multiple inhomogeneous tissue modifications, such as inflammation, dead or stressed tissue, our semi-automated segmentation method provides the overall percentage of unhealthy tissue of the entire organ and would, therefore, slightly overestimate the tumor margin and the tumorous regions volume. As tumor volume estimation plays an important role in lung research, tumor burden quantification should not be restricted to 2D-limited H&E histological studies. Since one single imaging technique cannot provide conclusive tumor volume evaluation, a more reliable quantification of tumors in *ex vivo* tumorous lungs will result from combining a 3D imaging technique like micro-CT with histology. Moreover, the method that we present here holds great potential to be explored as a 3D histopathology procedure if staining agents capable of specifically targeting the tumor cells are used.

## Supplementary information

Supplementary info

## Data Availability

The datasets used and/or analyzed during the current study are available from the corresponding author on reasonable request.
