# Supplementary material for: A step towards valid detection and quantification of lung cancer volume in experimental mice with contrast agent-based X-ray microtomography
Source: Sci Rep. 2019 Feb 4;9:1325. doi: 10.1038/s41598-018-37394-w (PMC6362109; doi:10.1038/s41598-018-37394-w)
Supplement: Supplementary file 1 — Supplementary info [file 41598_2018_37394_MOESM1_ESM.pdf]

A step towards valid detection and quantification of lung cancer volume in experimental mice with contrast agent-based X-ray microtomography

Pidassa Bidola,[a] Juliana Martins de Souza e Silva,[a,+,*] Klaus Achterhold,[a] Enkhtsetseg Munkhbaatar,[b] Philipp J. Jost,[b] Anna-Lena Meinhardt,[b] Kirsten Taphorn,[a] Marie-Christine Zdora,[c,d] Franz Pfeiffer,[a,e] and Julia Herzen[a]

[a] Chair of Biomedical Physics, Department of Physics & Munich School of Bioengineering, Technical University of Munich, 85748 Garching, Germany.

[b] III. Medizinische Klinik, Klinikum rechts der Isar, Technical University of Munich, 81675 Munich, Germany.

[c] Diamond Light Source, Harwell Science and Innovation Campus, Didcot, Oxfordshire, OX11 0DE, United Kingdom.

[d] Department of Physics & Astronomy, University College London, London, WC1E 6BT, United Kingdom.

[e] Department of Diagnostic and Interventional Radiology, Klinikum rechts der Isar, Technical University of Munich, 81675 Munich, Germany.

[+] Present address: Institute of Physics, Martin-Luther-University Halle-Wittenberg, 06120 Halle (Saale), Germany

Pidassa Bidola and Juliana Martins de Souza e Silva equally contributed to this work.

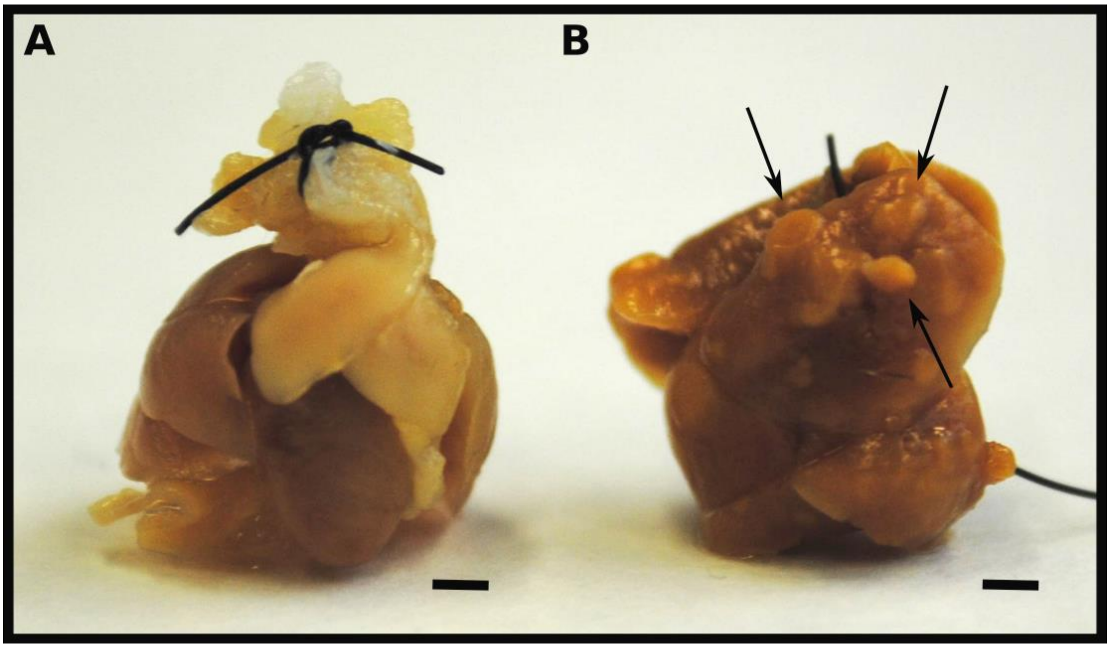

Figure S1. Digital pictures of (A) the control lung and (B) a tumorous lung before staining.

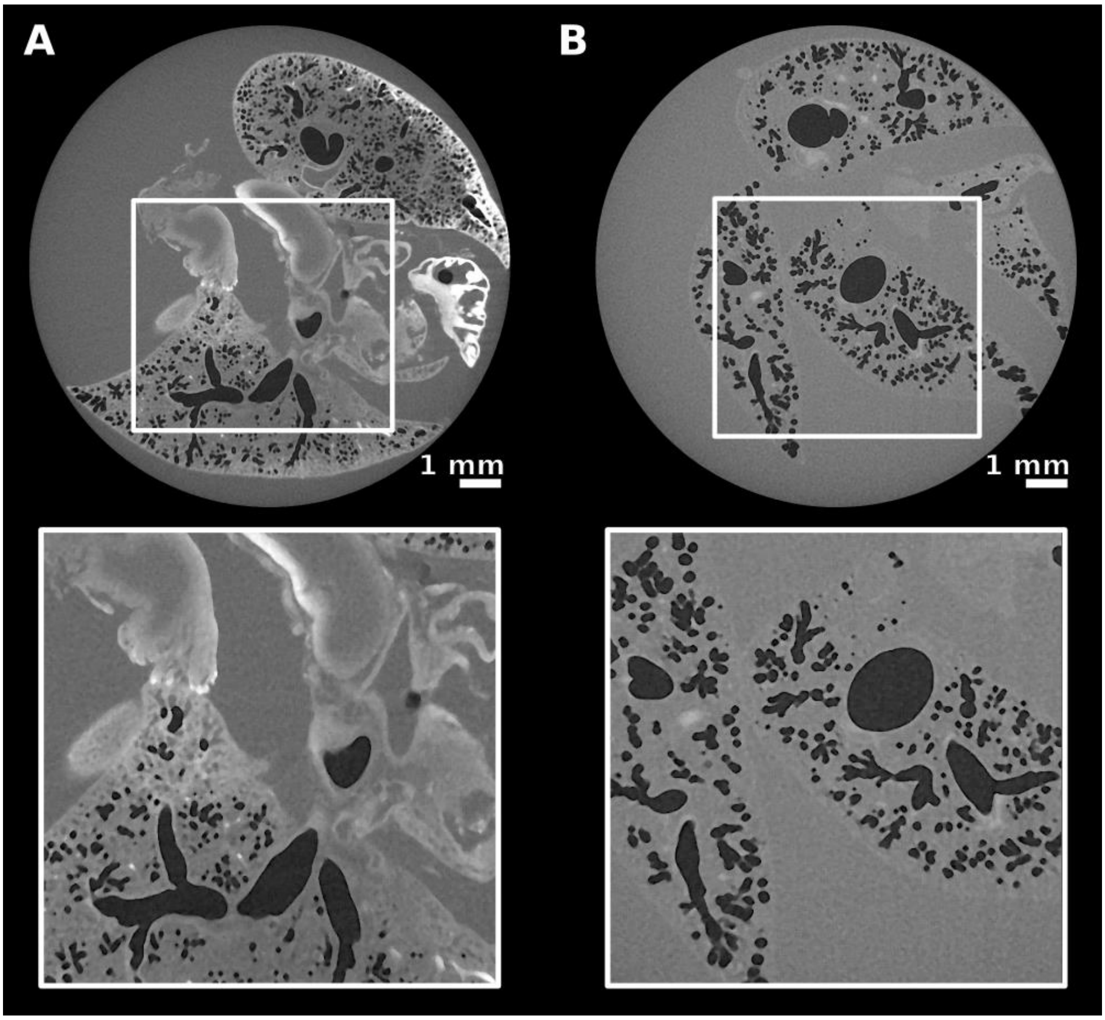

Figure S2. Tomograms of lungs stained (A) with phosphotungstic acid in water (PTA) and (B) with a mixture of iodine and potassium iodide (known as IKI).
